# Rapid Room-Temperature Preparation of Hierarchically Porous Metal–Organic Frameworks for Efficient Uranium Removal from Aqueous Solutions

**DOI:** 10.3390/nano10081539

**Published:** 2020-08-06

**Authors:** Chongxiong Duan, Yi Zhang, Jiexin Li, Le Kang, Yawei Xie, Wenxiong Qiao, Chunxia Zhu, Haochuan Luo

**Affiliations:** 1 School of Materials Science and Hydrogen Energy, Foshan University, Foshan 528231, China; cechxduan@mail.scut.edu.cn (C.D.); yaweideyouxiang@gmail.com (Y.X.); 2 School of Energy Sciences and Engineering, Nanjing Tech University, Nanjing 211816, China; 3 School of Chemistry and Chemical Engineering, University of South China, Hengyang 421001, China; ljx19980624@outlook.com (J.L.); qwenxiong512@outlook.com (W.Q.); zcx7842@gmail.com (C.Z.); 4 School of Materials Science and Engineering, Xi’an University of Science and Technology, Xi’an 710054, China; kangle20140805@126.com

**Keywords:** hierarchically porous Cu-BTC, facile synthesis, uranium (VI) removal, enhanced adsorption capacity

## Abstract

The effective removal of uranium from an aqueous solution is a highly valuable process for the environment and health. In this study, we developed a facile and rapid method to synthesize hierarchically porous Cu-BTC (RT-Cu-BTC) using a cooperative template strategy. The as-synthesized RT-Cu-BTC exhibited hierarchically porous structure and excellent thermostability, as revealed by X-ray powder diffraction, Fourier-transform infrared spectroscopy, scanning electron microscopy, and thermogravimetric analysis. Compared with conventional metal–organic frameworks (MOFs) and zeolites, the obtained RT-Cu-BTC exhibited enhanced adsorption capacity (839.7 mg·g^−1^) and high removal efficiency (99.8%) in the capture of uranium (VI) from aqueous solutions. Furthermore, the conditions such as adsorbent dose, contact time, and temperature in adsorption of uranium (VI) by RT-Cu-BTC were investigated in detail. The thermodynamics data demonstrated the spontaneous and endothermic nature of the uranium (VI) adsorption process. The Langmuir isotherm and pseudo-second-order models could better reflect the adsorption process of uranium (VI) onto RT-Cu-BTC. In addition, the as-synthesized RT-Cu-BTC showed excellent stability in removing uranium (VI) from an aqueous solution. This work provides a facile and rapid approach for fabricating hierarchically porous MOFs to realize a highly efficient removal of uranium (VI) from aqueous systems.

## 1. Introduction

As a representative radioactive element, uranium is a primary fuel that is used to produce nuclear power and has been widely used in various fields such as national defense and nuclear power plants [1,2]. However, uranium is harmful to the ecological environment and health due to its radioactivity and toxicity, even at trace concentrations [3]. Therefore, the development of effective methods for adsorption and removal of uranium from radioactive wastewater is of great significance. Over the past decades, available approaches such as chemical precipitation [4], solvent extraction [5], ion exchange [6], coagulation [7], and adsorption [8,9] have been developed to extract and remove uranium. Among them, adsorption is considered an effective method for the removal of uranium due to its simple operation process, high efficiency, and low cost, especially for use with low concentrations [10,11].

Hierarchically porous metal–organic frameworks (MOFs) that are self-assembled from organic ligands and metal ions (or clusters) have multimodal hierarchically porous structures with micropores and mesopores (or micropores, mesopores, and macropores) [12,13,14]. Compared with conventional microporous MOFs, mesopores and macropores in hierarchically porous MOFs can provide large channels to facilitate the diffusion and accessibility of guest molecules [15]. Meanwhile, micropores retain high surface area and guarantee abundant active sites [16,17]. However, the majority of MOFs are restricted to a microporous regime (pore size < 2 nm) [18]. In the past decades, microporous MOF materials have been widely used as adsorbents for adsorption of uranium (VI) from aqueous solutions [19]. For example, Wang et al. [20] reported that the adsorption capacity of uranium (VI) onto microporous Cu-BTC was 744 mg·g^−1^. Other conventional MOFs, such as UIO-66, UIO-68, and MOF-76 have also been applied as adsorbents for the removal of uranium (VI) [21,22,23]. However, to the best of our knowledge, there have been no reports on hierarchically porous MOF materials being used as adsorbents to remove uranium (VI) from an aqueous solution.

In this work, hierarchically porous Cu-BTC (RT-Cu-BTC) was synthesized successfully within 30 min under facile conditions. The product RT-Cu-BTC was sufficiently characterized by X-ray powder diffraction, Fourier-transform infrared spectroscopy, scanning electron microscopy, and thermogravimetric analysis. The resulting RT-Cu-BTC was used to adsorb uranium (VI) from aqueous solutions, and various parameters including adsorbent dose, contact time, and temperature were optimized. In addition, the adsorption behavior of uranium (VI) onto RT-Cu-BTC was investigated based on pseudo-first-order and pseudo-second-order models, as well as Langmuir and Freundlich models. The as-synthesized RT-Cu-BTC exhibited higher saturation uptake and removal efficiency of uranium (VI) as compared with conventional MOFs and zeolites.

## 2. Materials and Methods

### 2.1. Rapid Room-Temperature Preparation of Hierarchically Porous Cu-BTC

Hierarchically porous Cu-BTC was synthesized at room temperature and pressure within 30 min by following our previous method [13]. Specifically, 0.293 g of ZnO powder was added to 8 mL of deionized water to form a nanoslurry using sonication for 30 min, and then the nanoslurry was transferred into a 16 mL *N*,*N*-dimethylmethanamide (DMF) solvent (solution A). Subsequently, solution B was prepared by adding 7.2 mmol of copper nitrate trihydrate (Cu(NO_3_)_2_·3H_2_O) to 18 mL of deionized water; solution C was prepared by adding 4 mmol of 1,3,5-benzenetricarboxylic acid (H_3_BTC) and 14.4 mmol of 1-bromohexadecane to 16 mL of ethanol. After stirring for 30 min, solution A and solution B were mixed and continuously stirred for 10 min (solution D). Finally, two solutions (solution C and solution D) were combined and continuously stirred for 30 min at room temperature and pressure. The obtained precipitate was immediately filtered and immersed in ethanol solution at 373 K (four times), and then it was dried in an oven at 393 K overnight. The resulting product is denoted as RT-Cu-BTC.

### 2.2. Material Characterization

X-ray diffraction (XRD) patterns were obtained on a diffractometer system (D8 ADVANCE, Bruker AXS, Karlsruhe, Germany) by using Ni-filtered Cu-target K_α_ radiation (40 kV, 40 mA, wavelength *λ* = 0.15418 nm). The XRD patterns of Cu-BTC were simulated through the Materials Studio package 5.0 (CCDC: 112954). Fourier-transform infrared (FTIR) spectra of samples in the form of KBr pellets were obtained from an FTIR spectrometer (Vector 33, Bruker Corporation, Karlsruhe, Germany) with a resolution of 4 cm^−1^. The morphology analysis was examined by scanning electron microscopy (SEM; ZEISS Ultra 55, Carl Zeiss, Oberkochen, Germany). Thermogravimetric analysis (TGA) of the material was obtained from a TG analyzer (TG 209, Netzsch, Selb, Germany), and the sample was heated from 298 to 873 K in an N_2_ atmosphere at a rate of 5 K min^−1^.

### 2.3. Adsorption Experiments

As in a typical procedure [24], a certain amount of RT-Cu-BTC adsorbent and 150 mL of 35 mg/L uranium (VI) solution were first added to the conical flask (250 mL). Next, the conical flask was shaken using mechanical stirring for a specified time period at different temperatures. After shaking for a required time at a certain temperature, the adsorbent was separated by filtration and the remaining uranium (VI) concentration was analyzed using a UV-Vis spectrophotometer (details are shown in the Supporting Information (SI)). The removal efficiency (*η*) was calculated using the following equation:

$$\eta =\frac{C_{0}-C_{e}}{C_{0}}\times 100\%$$

where *C*_0_ and *C_e_* (mg/L) represent the concentration of U (VI) at the initial and equilibrium state, respectively.

## 3. Results and Discussion

### 3.1. Crystal Structure Identification of RT-Cu-BTC

**Figure 1:**
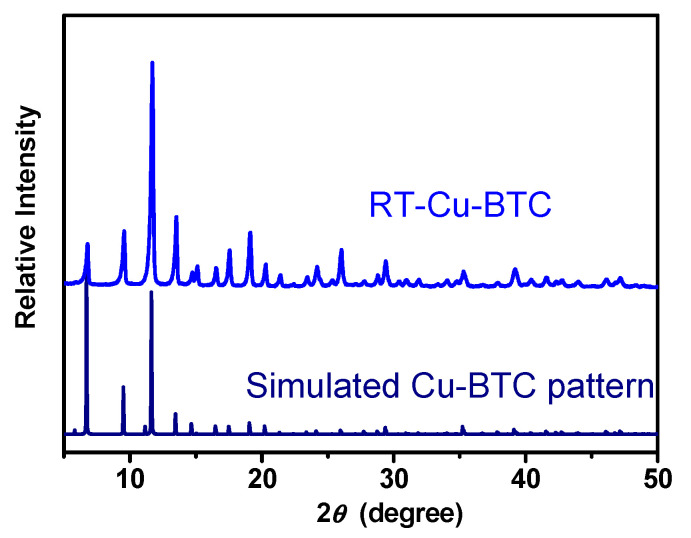
X-ray diffraction (XRD) patterns of the as-synthesized RT-Cu-BTC and the simulated Cu-BTC pattern.

The RT-Cu-BTC crystal XRD data were acquired using an X-ray diffractometer. As shown in Figure 1, the wide-angle XRD pattern of the RT-Cu-BTC is in agreement with the simulated pattern of Cu-BTC, indicating that the introduced hydroxy double salt (HDS) and template were removed by the washing and drying process after synthesis [13,25]. Moreover, the FTIR spectrum of RT-Cu-BTC exhibits four characteristic absorption bands located at approximately 1600, 1560, 1450, and 1370 cm^−1^ (Figure S1), consistent with the previous reports of conventional Cu-BTC [26]. These results indicate that the attained RT-Cu-BTC sample possesses a framework connection identical to that of conventional Cu-BTC [27]. Unlike the conventional Cu-BTC particle with an octahedral morphology and smooth surface (Figure S2a), the SEM image in Figure S2b displays that the as-synthesized RT-Cu-BTC particle has a basic octahedral shape and a large number of worm-like pores distributed on the surface of the crystals. TGA indicates that the attained RT-Cu-BTC is stable at temperatures up to 320 °C (Figure S3), which agrees well with the conventional Cu-BTC. In addition, our previous work confirms the formation of mesopores in the RT-Cu-BTC sample [13], as shown in Table S1 and Figure S4. These results indicate the successful fabrication of stable hierarchically porous Cu-BTC material under facile conditions. Compared to the conventional solvothermal method for synthesizing hierarchically porous MOFs with harsh processing conditions (e.g., high temperature and long crystallization times) [28,29], the present method requires much less energy; it is also simple, much safer, and environmentally friendly, and it may be readily scaled up.

### 3.2. The Removal of Uranium (VI) by RT-Cu-BTC from Aqueous Solutions

#### 3.2.1. Effect of RT-Cu-BTC Dosage

**Figure 2:**
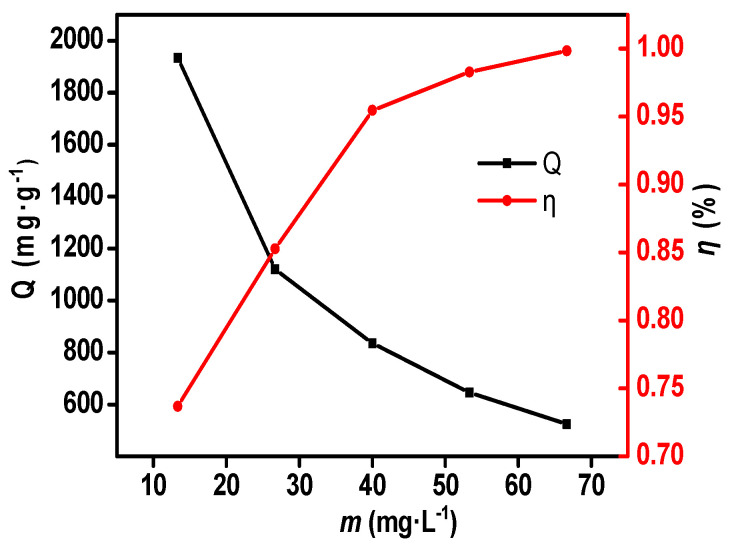
Influence of RT-Cu-BTC dosage on removal efficiency and adsorption capacity.

The influence of adsorbent dosage on the adsorption of uranium (VI) was investigated by varying the amount of RT-Cu-BTC while other parameters (contact time and temperature) were maintained as constants (see Supporting Information for details). As shown in Figure 2, the removal efficiency of uranium (VI) dramatically increased with the dosage of adsorbent. This is due to the fact that more active sites for uranium adsorption were provided with the increase of additional amounts of adsorbent [30]. With a further increase in the adsorbent dosage, the removal efficiency of uranium (VI) exhibited a slowly increasing trend, with the maximum value reaching 99.8%. This result could be attributed to the active sites of the adsorbent reaching saturation when the amount of RT-Cu-BTC increased to a certain extent [24]. It should be noted, however, that the adsorption capacity of uranium (VI) onto the RT-Cu-BTC always decreased with the increase of adsorbent, most likely because the amount of uranium (VI) adsorbed per unit mass of adsorbent decreased with the increase of the adsorbent dosage [31].

#### 3.2.2. Effect of Contact Time

**Figure 3:**
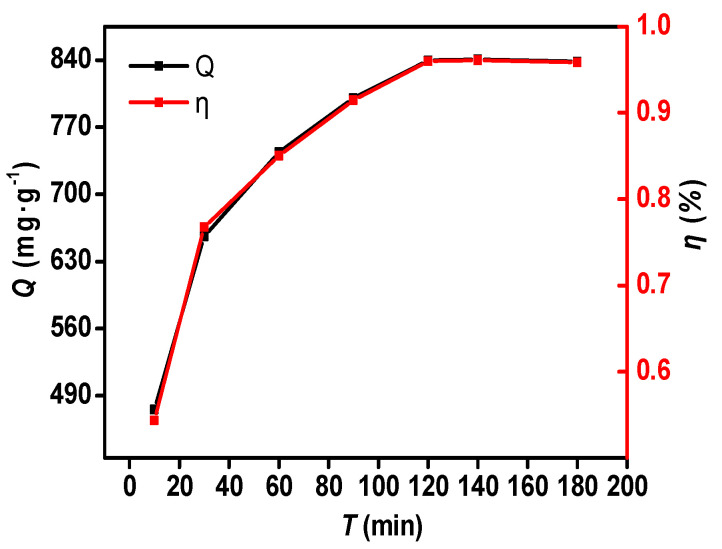
Influence of contact time on the adsorption process.

Figure 3 shows the adsorption isotherms of uranium (VI) on the RT-Cu-BTC adsorbent at different contact times. As shown in Figure 3, the adsorption curves showing the uptake of uranium (VI) by RT-Cu-BTC exhibited three different stages. During the first 30 min, the uptake of uranium (VI) by RT-Cu-BTC increased rapidly with an increase of contact time, and a large amount of uranium (VI) was removed, which can be attributed to the higher concentration gradient of uranium (VI) and more available active sites of adsorbent during this period [30]. Subsequently, the adsorption capacity of RT-Cu-BTC showed a steadily increasing trend within 30–120 min as the active sites of the adsorbent gradually reached saturation [32]. After 120 min, the adsorption reached equilibrium, and the maximum adsorption capacity and adsorption efficiency were 839.7 mg·g^−1^ and 99.8%, respectively. The high capacity and efficiency in adsorption of uranium (VI) can be attributed to the high Brunauer–Emmett–Teller (BET) surface area and large pore volume of RT-Cu-BTC [33]. These results indicate that the adsorption equilibrium of uranium (VI) adsorption by RT-Cu-BTC can be reached within a short time.

#### 3.2.3. Effect of Temperature and Adsorption Thermodynamics

**Figure 4:**
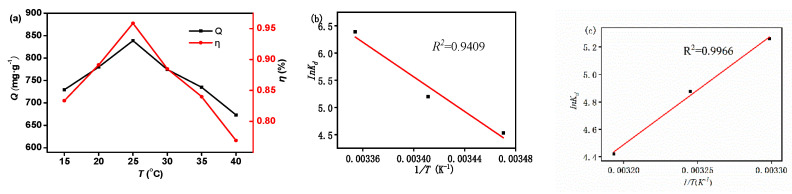
(**a**) Adsorption isotherms of RT-Cu-BTC for uranium (VI) at different temperatures; (**b**) van ’t Hoff plot for the removal of uranium (VI) by RT-Cu-BTC adsorbent; and (**c**) fit line between In*K_d_* and 1/T (30–45 °C).

The effect of temperature on adsorption behavior of the RT-Cu-BTC adsorbent is shown in Figure 4a. The adsorption capacity and adsorption efficiency of uranium (VI) uptake by RT-Cu-BTC increased as temperature increased from 15 to 25 °C, indicating the endothermic nature of the adsorption process [34]. However, as the temperature was further increased, the adsorption capacity and adsorption efficiency decreased rapidly. This is attributed to the interaction between the active sites of adsorbent and the uranium (VI) being weaker at higher temperatures [30]. Therefore, the optimal temperature for adsorption of uranium (VI) on the RT-Cu-BTC is 25 °C.

The Gibbs free energy change (Δ*G*, KJ·mol^−1^) is a fundamental parameter for estimating the spontaneity of a reaction [35]. The Δ*G* was calculated using the Gibbs–Helmholtz Equation (1) [36]:

(1) ΔG=ΔH−TΔS

where Δ*H* is the enthalpy change (KJ·mol^−1^), Δ*S* is the entropy change (J·mol^−1^·K^−1^), and *T* is the temperature (K). The values of Δ*H* and Δ*S* were obtained from the van ’t Hoff plot (Figure 4b,c), and the corresponding thermodynamics parameters are summarized in Table 1 [37]. The positive value of Δ*H* (132.329 KJ·mol^−1^) indicates that the adsorption process of uranium (VI) on the RT-Cu-BTC was endothermic, which is consistent with the previous reports [20]. The positive value of Δ*S* (496.17 J·mol^−1^·K^−1^) can be attributed to the increased degree of randomness during the adsorption of uranium (VI) on the active sites of RT-Cu-BTC [38]. The value of Δ*G* at all temperatures was negative, which indicates that the adsorption process was feasible and spontaneous thermodynamically. Since the value of Δ*G* decreased with the increase of temperature, it can be known that the adsorption of uranium (VI) onto RT-Cu-BTC was favorable at higher temperatures [3], which is consistent with the adsorption isotherms (Figure 4a). Moreover, when *T* = 30–45 °C, the value of Δ*H* was negative (Table S2), indicating that the adsorption was essentially an exothermic process. The negative value of Δ*S* reveals the decrease in the degree of freedom of the process. At the same time, at the four different temperatures, the Gibbs free energy was less than 0, indicating that the adsorption of uranium (VI) by RT-Cu-BTC was an unspontaneous process, in which the adsorption capacity of RT-Cu-BTC for uranium (VI) was greatly reduced. In addition, the value of |Δ*H*| was always greater than |*T*Δ*S*|, indicating that the adsorption process was dominated by enthalpy rather than entropy changes [30].

### 3.3. Adsorption Kinetics

**Figure 5:**
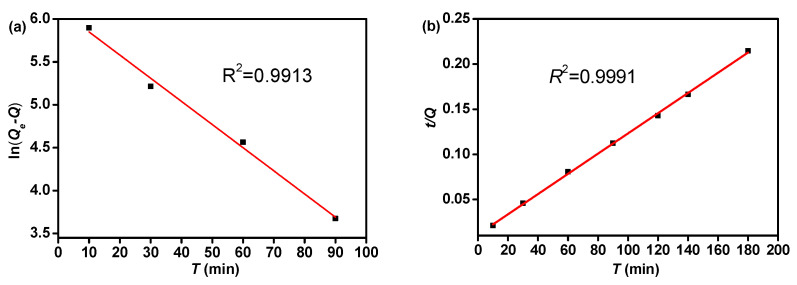
The simulation curves of (**a**) pseudo-first-order and (**b**) pseudo-second-order kinetic models for adsorption of uranium (VI) by RT-Cu-BTC adsorbent.

Two models, namely pseudo-first-order and pseudo-second-order models, were applied to investigate the adsorption kinetics of uranium (VI) on RT-Cu-BTC, and the corresponding equations are expressed as follows [39]:

(2) $$In(q_{e}-q_{t})=Inq_{e}-k_{1}t\;\text{Pseudo-first-order}\;\text{equation}$$

(3) $$\frac{t}{q_{t}}=\frac{1}{k_{2}q_{e}^{2}}+\frac{t}{q_{e}}\;\text{Pseudo-second-order}\;\text{equation}$$

where *q_e_* and *q_t_* represent the equilibrium adsorption capacity and the specific adsorption capacity at a certain time (mol·g^−1^), respectively; *K*_1_ (min^−1^) and *K*_2_ (g·mol^−1^·min^−1^) are the rate constants of the pseudo-first order and the pseudo-second order, respectively. The correlation coefficient (*R*^2^) and rate constants of the two models are shown in Figure 5 and Table 2. As shown in Figure 5, the correlation coefficient obtained from the pseudo-second-order model (*R*^2^ = 0.9991) was higher than that of the pseudo-first-order model (*R*^2^ = 0.9913), and the value of *q_e_* (1000 mg·g^−1^) obtained from the pseudo-second-order model is close to the experimental value (839.7 mg·g^−1^), as shown in Table 2. These results indicate that the adsorption of uranium (VI) on RT-Cu-BTC adsorbent follows the pseudo-second-order model better and that the corresponding adsorption process might be chemical adsorption [33].

### 3.4. Adsorption Isotherms

**Figure 6:**
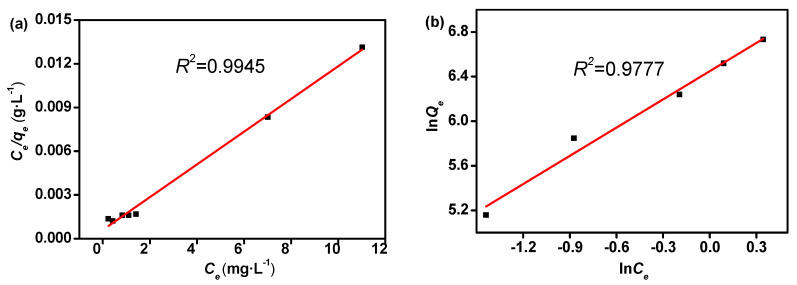
(**a**) Langmuir adsorption isotherm and (**b**) Freundlich adsorption isotherm.

To describe the behavior of the adsorption of uranium (VI) onto RT-Cu-BTC, the Langmuir and Freundlich models were used to simulate the adsorption process [40]. The two models were calculated from the following equations [30]:

(4) $$\frac{C_{e}}{q_{e}}=\frac{C_{e}}{Q_{m}}+\frac{1}{K_{L}Q_{m}}\;\text{Langmuir}\;\text{equation}$$

(5) $$lnq_{e}=lnK_{F}+\frac{1}{n}lnC_{e}\;\text{Freundlich}\;\text{equation}$$

where *Q_m_* (mg·g^−1^) and *q_e_* (mg·g^−1^) are the maximum and equilibrium adsorption capacities of uranium (VI), respectively; *C_e_* (mg·L^−1^) is the equilibrium concentration of uranium (VI); *K_L_* (L·mg^−1^) and *K_F_* (L·mg^−1^) are the Langmuir and Freundlich constants; and *1/n* is the adsorption intensity. The fitting isotherms of all the adsorption data and corresponding parameters are shown in Figure 6 and Table 3. As shown in Figure 6, the correlation coefficient (*R*^2^) obtained from the Langmuir isotherm model (*R*^2^ = 0.9945) was slightly higher than that of Freundlich (*R*^2^ = 0.9777), indicating that the Langmuir isotherm model fits quite well with the experimental data with regard to uranium (VI) adsorption on RT-Cu-BTC. This implies that the adsorption of uranium (VI) by Cu-BTC adsorbent was a monolayer adsorption process [40], and this finding is in agreement with a similar conclusion drawn in the earlier work of Feng et al. [20]. As observed in Table 3, the value of *Q_m_* obtained from the Langmuir model was 892.9 mg·g^−1^, which is close to the experimental result (*q_e_* = 839.7 mg·g^−1^). The value of *K_f_* demonstrates that the adsorption capacity of uranium (VI) on RT-Cu-BTC adsorbent increased with an increase of temperature from 15 to 25 °C, which is consistent with the results of the Langmuir model and adsorption isotherm (Figure 4a) [41]. Furthermore, the value of 1 < *n*(1.19) < 10 indicates the favorable adsorption of uranium (VI) onto RT-Cu-BTC adsorbent [20].

### 3.5. Crystal Structure of RT-Cu-BTC after Adsorption of Uranium (VI)

**Figure 7:**
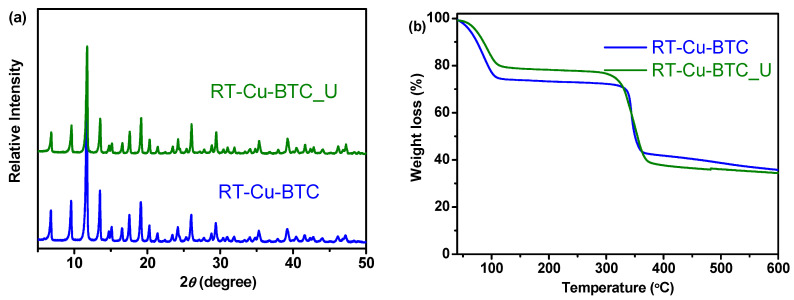
(**a**) Powder XRD patterns and (**b**) thermogravimetric analysis (TGA) of Cu-BTC before (RT-Cu-BTC) and after (RT-Cu-BTC_U) uranium (VI) adsorption.

The crystal structure of RT-Cu-BTC (denoted as RT-Cu-BTC_U) after adsorption of uranium (VI) was investigated in detail. As shown in Figure 7a, the diffraction peak positions and relative intensities of the RT-Cu-BTC_U are in accord with the RT-Cu-BTC, indicating that the absorbed uranium (VI) has no influence on the framework structure of crystals. As observed in Figure 7b, the RT-Cu-BTC_U sample shows a weight change curve similar to that of RT-Cu-BTC, indicating that the thermal stability of hierarchically porous Cu-BTC materials can be maintained after the adsorption of uranium (VI). In addition, the SEM image further reveals that the RT-Cu-BTC_U sample maintains the original near-octahedral morphology (Figure S5). These results indicate that hierarchically porous Cu-BTC material possesses excellent stability for the adsorption of uranium (VI) from an aqueous solution.

### 3.6. A Possible Mechanism for Adsorption of Uranium (VI) by RT-Cu-BTC

**Scheme 1:**
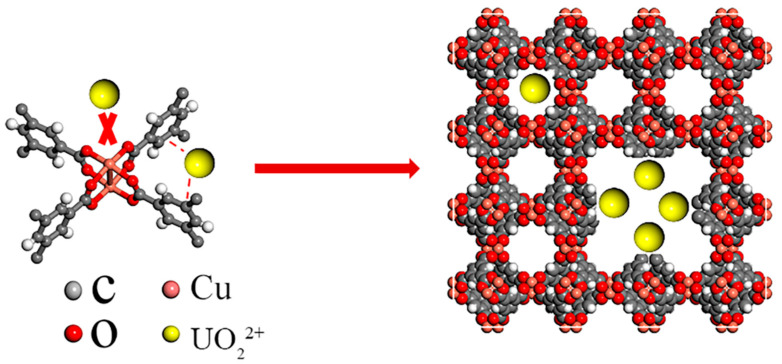
Map of possible mechanism for the adsorption of uranyl ion (UO_2_^2+^) by RT-Cu-BTC.

According to previous literature [42,43,44], although the framework of Cu-BTC was electrically neutral, some negative charges localized on the carboxyl groups owing to the presence of abundant oxygen atoms in these carboxylate units, which provides possible coordination sites for uranyl ions (UO_2_^2+^) with positive charges [45]. These results indicate that the adsorption of UO_2_^2+^ onto RT-Cu-BTC depends not only on the coordination interaction, but also on the Coulomb electrostatic interaction. Therefore, a possible mechanism for adsorption of UO_2_^2+^ by RT-Cu-BTC is illustrated in Scheme 1. In the initial stage, the RT-Cu-BTC was highly dispersed in aqueous solutions due to the high affinity of Cu^2+^ sites to water [46]. Then, the UO_2_^2+^ with positive charges aggregated around the carboxyl groups due to the coordination interaction and Coulomb electrostatic interaction [20]. By contrast, the UO_2_^2+^ is rarely distributed around the Cu^2+^ site owing to the repulsion between like charges and steric hindrance [47,48].

### 3.7. The Enhanced Adsorption Capacity of Uranium (VI)

Table 4 summarizes the maximum adsorption capacity of uranium (VI) on various adsorbents. As shown in Table 4, the uptake of uranium (VI) by RT-Cu-BTC was 839.7 mg·g^−1^, which is higher than the uptakes of a series of MIL-101 (20 mg·g^−1^), UIO-66 (109.9 mg·g^−1^), and Zn-MOF materials and also easily surpasses the conventional Cu-BTC (744 mg·g^−1^). The enhanced uranium (VI) adsorption capacity of RT-Cu-BTC can be attributed to the introduction of mesoporous structure [20,33]. In addition, RT-Cu-BTC exhibited significantly higher saturation uptake of uranium (VI) than those of conventional crystalline materials, such as metal oxide and zeolites (Table 4). This was probably due to the high BET surface area and large pore volume of RT-Cu-BTC (Table S1) [8,49]. These results indicate that hierarchically porous Cu-BTC material has an excellent adsorption capacity for the removal of uranium (VI) from an aqueous solution.

## 4. Conclusions

In summary, hierarchically porous Cu-BTC (RT-Cu-BTC) material was successfully synthesized under facile conditions within 30 min using a cooperative template strategy. Compared with MIL-101, UIO-66, Zn-MOFs, and Cu-BTC, the as-synthesized RT-Cu-BTC exhibited significantly enhanced capacity (839.7 mg·g^−1^) in adsorbing and removing uranium (VI) from aqueous solutions. The high adsorption capacity of RT-Cu-BTC can be attributed to the introduction of hierarchically porous structures. By investigating the amount of RT-Cu-BTC (*m*), contact time (*t*), and temperature (*T*), the optimal conditions (*m* = 6 mg, *t* = 120 min, *T* = 25 °C) for uranium (VI) adsorption were obtained. In addition, the thermodynamics data indicate that the adsorption process of uranium (VI) onto RT-Cu-BTC was feasible and spontaneous. The pseudo-second-order model can better reflect the adsorption kinetics, and the Langmuir adsorption isotherm model may help understand the adsorption process of uranium (VI) on RT-Cu-BTC. These results indicate that hierarchically porous MOF materials are highly efficient adsorbents for the removal of uranium from aqueous solutions owing to their hierarchically porous structures.

## Figures and Tables

**Table 1:** Thermodynamic parameters for adsorption of uranium (VI) by RT-Cu-BTC at 15–25 °C.

Δ*H* [KJ·mol^−1^]	Δ*S* [J·mol^−1^·K^−1^]		Δ*G* [KJ·mol^−1^]	
		288.15 K	293.15 K	298.15 K
132.329	496.17	−10.643	−13.123	−15.604

**Table 2:** Kinetic Models Constants for the Adsorption of Uranium (VI) by RT-Cu-BTC.

Pseudo-First-Order			Pseudo-Second-Order			*q_e_* (exp) [mg·g^−1^]
*K*_1_ [min^−1^]	*q_e_* [mg·g^−1^]	*R* ^2^	*K*_2_ [g·mg^−1^·min^−1^]	*q_e_* [mg·g^−1^]	*R* ^2^	
−0.027	454.45	0.9913	0.0001	1000	0.9991	839.7

**Table 3:** Parameters for adsorption of uranium (VI) on RT-Cu-BTC.

*T* (°C)	Langmuir			Freundlich		
	*Q_m_* [mg·g^−1^]	*K_L_* [L·mg^−1^]	*R* ^2^	*K_F_* [mg^1−n^·L^n^·g^−1^]	*n*	*R* ^2^
25	892.9	1.86	0.9945	631.4	1.19	0.9777

**Table 4:** Saturation adsorption capacity of uranium (VI) on various adsorbents.

No	Adsorbent	Temperature [K]	Uranium Capacity [mg·g^−1^]	Ref.
1	MIL-101	298	20	[50]
2	MIL-101-NH_2_	298	90	[50]
3	MIL-101-ED	298	200	[50]
4	MIL-101-DETA	298	350	[50]
5	Zn-MOF	298	115	[19]
6	UIO-66	-	109.9	[21]
7	UIO-66-NH_2_	-	114.9	[21]
8	UIO-68	295	217	[48]
9	Zn-MOF-74	298	360	[51]
10	MOF-76	288	300	[23]
11	Cu-BTC	298	744	[20]
12	Fe_3_O_4_@Zn–Al–LDHs	-	36.9	[52]
13	SBA-15	298	409	[53]
14	RT-Cu-BTC	298	839.7	This work

## Supplementary Materials

The following are available online at https://www.mdpi.com/2079-4991/10/8/1539/s1, Table S1: Porosity properties of C-Cu-BTC and RT-Cu-BTC sample, Table S2:Thermodynamic parameters for adsorption of uranium (VI) by RT-Cu-BTC at 30–40 °C, Figure S1: FTIR spectra of conventional Cu-BTC (C-Cu-BTC) and RT-Cu-BTC samples, Figure S2: SEM images of (a) C-Cu-BTC and (b) RT-Cu-BTC samples, Figure S3: Thermogravimetric analysis (TGA) curves of C-Cu-BTC and (b) RT-Cu-BTC samples, Figure S4: The N2 adsorption–desorption isotherms of RT-Cu-BTC sample, Figure S5: SEM image of RT-Cu-BTC_U sample
