# Supplementary material for: Rapid Room-Temperature Preparation of Hierarchically Porous Metal–Organic Frameworks for Efficient Uranium Removal from Aqueous Solutions
Source: Nanomaterials (Basel). 2020 Aug 6;10(8):1539. doi: 10.3390/nano10081539 (PMC7466529; doi:10.3390/nano10081539)
Supplement: Supplementary file 1 [file nanomaterials-10-01539-s001.pdf]

Rapid room-temperature preparation of hierarchically porous metal–organic frameworks for efficient uranium removal from aqueous solutions

Experimental section

Chemical reagent

Copper (II) nitrate trihydrate ($Cu(NO_3)_2 \cdot 3H_2O$, 99 %), 1,3,5-benzenetricarboxylic acid (H_3BTC, 99 %), zinc oxide (ZnO, 99 %), 1-bromohexadecane ($C_{16}H_{33}Br$, 98 %), uranium nitrate hydrate ($UO_2(NO_3)_2 \cdot 6H_2O$, AR). All of above chemical reagents used without further purification.

The adsorption of uranium (VI) on different dosages of RT-Cu-BTC

In a typical procedure, 150 mL of 35 mg/L uranium (VI) solution was weighed separately in 250 mL Erlenmeyer flasks numbered 1-5, and the pH of the uranium solution was adjusted to 7 by the addition of 0.1 M HNO_3 and 0.1 M NaOH. The RT-Cu-BTC adsorbents with masses of 2, 4, 6, 8, and 10 mg were respectively placed in the above-mentioned conical flask and mixed with the uranium (VI) solution, and the adsorbent was uniformly dispersed in the uranium (VI) solution by ultrasonic waves to ensure the adsorption efficiency. The flask was then placed in an oscillator at 25 ℃ for 180 min. After the adsorption process, the mixed solution was centrifuged, and 500 uL of the supernatant and 500 uL of arsenazo III were placed in a 5 mL volumetric flask and made up to volume with a buffer solution (chloroacetic acid, sodium acetate). The absorbance of the solution was measured at 652 nm using a UV VIS spectrophotometer.

The adsorption of uranium (VI) on RT-Cu-BTC with different contact times

In a typical procedure, 150 mL of 35 mg/L uranium (VI) solution were weighed separately in 250 mL Erlenmeyer flasks numbered 1-7, and the pH of the uranium solution was adjusted to 7 by the addition of 0.1 M HNO_3 and 0.1 M NaOH. After 6 mg of the RT-Cu-BTC adsorbent was separately added to the above-mentioned Erlenmeyer flask, the adsorbent was uniformly dispersed in the uranium solution by ultrasonic waves to ensure the adsorption efficiency. The Erlenmeyer flask was then placed in an oscillator with a contact time set to 10, 30, 60, 90, 120, 150, 180 min and a temperature of 25 ℃ to effect adsorption. After the adsorption process, the mixed solution was centrifuged, and 500 uL of the supernatant and 500 uL of arsenazo III were placed in a 5 mL

volumetric flask and made up to volume with a buffer solution (chloroacetic acid, sodium acetate). The absorbance of the solution was measured at 652 nm using a UV VIS spectrophotometer.

The adsorption of uranium (VI) on RT-Cu-BTC at different temperatures

In a typical procedure, 150 mL of 35 mg/L uranium (VI) solution were weighed separately in 250 mL Erlenmeyer flasks numbered 1-7, and the pH of the uranium solution was adjusted to 7 by the addition of 0.1 M HNO_3 and 0.1 M NaOH. After 6 mg of the RT-Cu-BTC adsorbent was separately added to the above-mentioned Erlenmeyer flask, the adsorbent was uniformly dispersed in the uranium solution by ultrasonic waves to ensure the adsorption efficiency. The Erlenmeyer flask was then placed in an oscillator with a temperature set to 15, 20, 25, 30, 35, 40 °C and a contact time of 180 min to effect adsorption. After the adsorption process, the mixed solution was centrifuged, and 500 uL of the supernatant and 500 uL of arsenazo III were placed in a 5 mL volumetric flask and made up to volume with a buffer solution (chloroacetic acid, sodium acetate). The absorbance of the solution was measured at 652 nm using a UV VIS spectrophotometer.

Table S1. Porosity properties of C-Cu-BTC and RT-Cu-BTC samples (data from Ref [1]).

Sample	S_{BET} [$m^2 \cdot g^{-1}$][a]	V_{total} [$cm^3 \cdot g^{-1}$][b]	V_{meso} [$cm^3 \cdot g^{-1}$][c]	V_{micro} [$cm^3 \cdot g^{-1}$][d]
C-Cu-BTC	1577	0.60	0.09	0.51
RT-Cu-BTC	869	0.58	0.21	0.37

[a] BET surface area; [b] Total pore volume; [c] Mesopore volume; [d] Micropore volume.

Table S2. Thermodynamic parameters for adsorption of uranium (VI) by RT-Cu-BTC at 30-40 °C.

ΔH [KJ mol^{-1}]	ΔS [J mol^{-1} K^{-1}]	ΔG [KJ mol^{-1}]		
		303.15 K	308.15 K	313.15 K
-66.086	-174.16	52.731	53.601	54.472

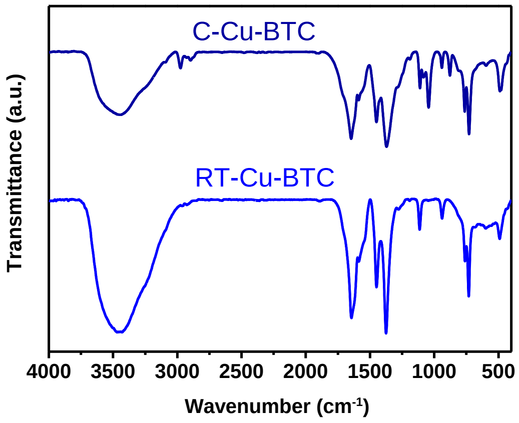

Figure S1. FTIR spectra of conventional Cu-BTC (C-Cu-BTC) and RT-Cu-BTC samples.

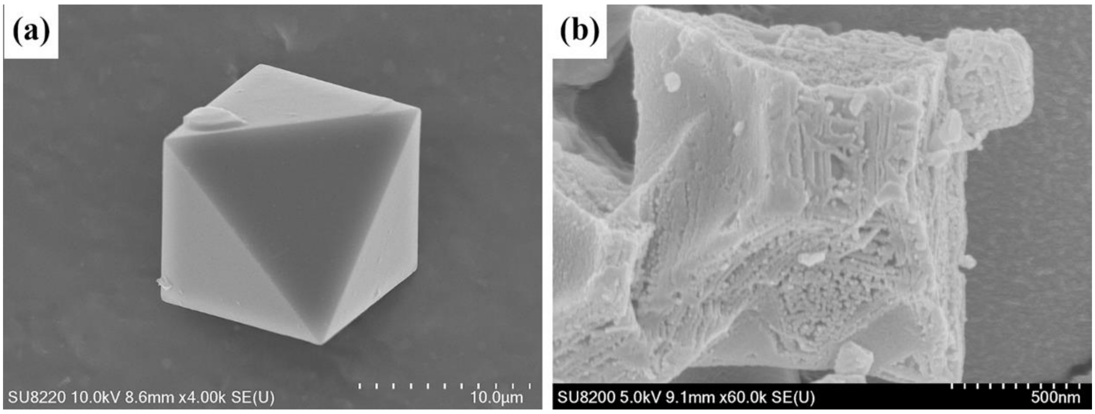

Figure S2. SEM images of (a) C-Cu-BTC and (b) RT-Cu-BTC samples.

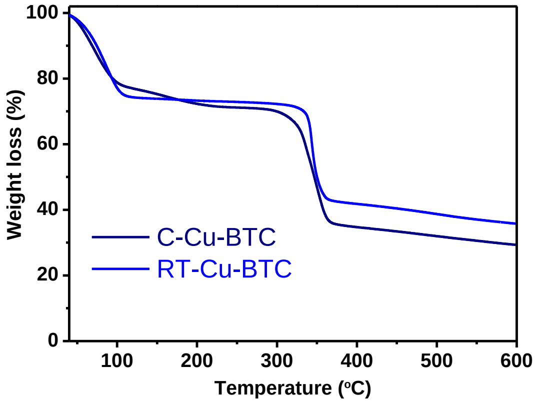

Figure S3. Thermogravimetric analysis (TGA) curves of C-Cu-BTC and (b) RT-Cu-BTC samples.

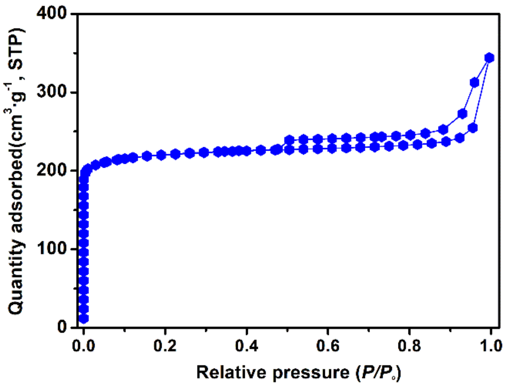

Figure S4. The N_2 adsorption–desorption isotherms of RT-Cu-BTC sample (data from Ref [1]).

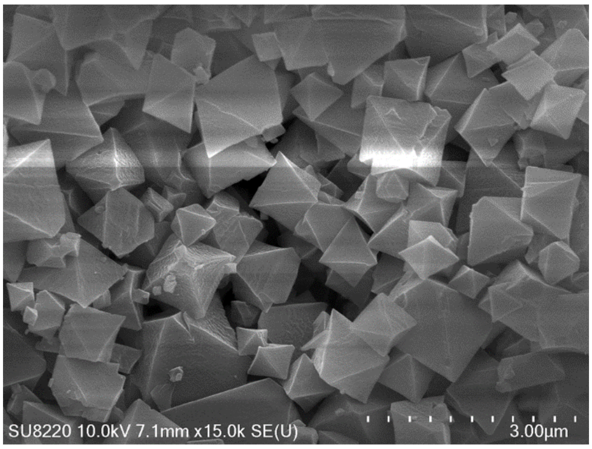

Figure S5. SEM image of RT-Cu-BTC_U sample.

Reference

1. Duan, C.; Li, F.; Li, L.; Zhang, H.; Wang, X.; Xiao, J.; Xi, H. Hierarchically structured metal-organic frameworks assembled by hydroxy double salt-template synergy with high space-time yields. *CrystEngComm* **2018**, *20*, 1057-1064.
